# Multiple phosphorylation sites regulate the activity of the repressor Mig1 in *Candida albicans*

**DOI:** 10.1128/msphere.00546-23

**Published:** 2023-11-27

**Authors:** Bernardo Ramírez-Zavala, Darina Betsova, Sonja Schwanfelder, Ines Krüger, Austin Mottola, Thomas Krüger, Olaf Kniemeyer, Axel A. Brakhage, Joachim Morschhäuser

**Affiliations:** 1 Institute of Molecular Infection Biology, University of Würzburg, Würzburg, Germany; 2 Department of Molecular and Applied Microbiology, Leibniz Institute for Natural Product Research and Infection Biology (HKI), Jena, Germany; 3 Institute of Microbiology, Friedrich Schiller University Jena, Jena, Germany; University of Georgia, Athens, Georgia, USA

**Keywords:** *Candida*, SNF1, Mig1

## Abstract

The highly conserved heterotrimeric protein kinase SNF1 is important for metabolic adaptations in the pathogenic yeast *Candida albicans*. A key function of SNF1 is to inactivate the repressor protein Mig1 and thereby allow the expression of genes that are required for the utilization of alternative carbon sources when the preferred carbon source, glucose, is absent or becomes limiting. However, how SNF1 controls Mig1 activity in *C. albicans* has remained elusive. Using a phosphoproteomics approach, we found that Mig1 is phosphorylated at multiple serine residues. Replacement of these serine residues by nonphosphorylatable alanine residues strongly increased the repressor activity of Mig1 in cells lacking a functional SNF1 complex, indicating that additional protein kinases are involved in the regulation of Mig1. Unlike wild-type Mig1, whose levels strongly decreased when the cells were grown on sucrose or glycerol instead of glucose, the levels of a mutant Mig1 protein lacking nine phosphorylation sites remained high under these conditions. Despite the increased protein levels and the absence of multiple phosphorylation sites, cells with a functional SNF1 complex could still sufficiently inhibit the hyperactive Mig1 to enable wild-type growth on alternative carbon sources. In line with this, phosphorylated forms of the mutant Mig1 were still detected in the presence and absence of a functional SNF1, demonstrating that Mig1 contains additional, unidentified phosphorylation sites and that downstream protein kinases are involved in the control of Mig1 activity by SNF1.

**IMPORTANCE:**

The SNF1 protein kinase signaling pathway, which is highly conserved in eukaryotic cells, is important for metabolic adaptations in the pathogenic yeast *Candida albicans*. However, so far, it has remained elusive how SNF1 controls the activity of one of its main effectors, the repressor protein Mig1 that inhibits the expression of genes required for the utilization of alternative carbon sources when glucose is available. In this study, we have identified multiple phosphorylation sites in Mig1 that contribute to its inactivation. Mutation of these sites strongly increased Mig1 repressor activity in the absence of SNF1, but SNF1 could still sufficiently inhibit the hyperactive Mig1 to enable growth on alternative carbon sources. These findings reveal features of Mig1 that are important for controlling its repressor activity. Furthermore, they demonstrate that both SNF1 and additional protein kinases regulate Mig1 in this pathogenic yeast.

## INTRODUCTION

Metabolic adaptations are important for the ability of the opportunistic fungal pathogen *Candida albicans* to colonize and grow in different host niches, in which the availability of nutrients may change (1). The heterotrimeric protein kinase SNF1, which consists of the catalytic α-subunit Snf1, the regulatory γ-subunit Snf4, and one of the two β-subunits Kis1 and Kis2, plays a key role in the utilization of alternative carbon sources when the preferred carbon source glucose is absent or becomes limiting (2, 3). The SNF1 complex is highly conserved in eukaryotes and has been studied extensively in the model organism *Saccharomyces cerevisiae* (4). In this yeast, a major function of SNF1 in metabolic adaptation is to phosphorylate and thereby inactivate the repressor protein Mig1, which together with the functionally related repressor Mig2 inhibits the expression of genes that are required for growth on alternative carbon sources when sufficient glucose is available (5–10).

*C. albicans* possesses homologs of both Mig1 and Mig2, which have largely overlapping functions in the downregulation of glucose-repressed genes (11). However, Mig1 lacks the consensus Snf1 phosphorylation sites, which has led to the hypothesis that SNF1 may control gene expression in a different way in this pathogenic yeast (12). Nevertheless, deletion of *MIG1* (and *MIG2*) alleviates the growth defects of *snf1*Δ and *snf4*Δ mutants, suggesting that Mig1 is a downstream target of SNF1 also in *C. albicans* (11, 13). Mig1 was found to be phosphorylated even in cells grown in YPD medium, which contains glucose as the main carbon source (14). Mig1 remained phosphorylated in *snf1*Δ and *snf4*Δ mutants, although the migration pattern of HA-tagged Mig1 detected in western blots was altered, indicating that SNF1 may regulate the repressor indirectly (14). Phosphorylated forms of Mig1 were also detected after shifting the cells from glucose to alternative carbon sources, but the total amount of the protein was reduced without changes in *MIG1* mRNA levels, indicating protein depletion as a mechanism of Mig1 inactivation (11, 14). A previous large-scale phosphoproteome analysis of *C. albicans* grown under various conditions did not identify phosphopeptides derived from Mig1 (15). So far, at which sites Mig1 is phosphorylated and how this influences its repressor activity have remained elusive.

In our present study, we harnessed data from a phosphoproteome analysis of the *C. albicans* wild-type strain SC5314 and various protein kinase deletion mutants performed by our groups (unpublished results), which allowed us to identify phosphorylation sites in Mig1. We therefore set out to assess how Mig1 phosphorylation at these sites affects its activity as a repressor protein.

## RESULTS

### Identification of Mig1 phosphorylation sites

In a phosphoproteome analysis of the *C. albicans* wild-type strain SC5314 and various protein kinase deletion mutants grown to log phase at 30°C in YPD medium without or with an iron chelator (our unpublished results), several phosphopeptides were identified that could be assigned to the Mig1 protein (Table 1; see Table S1 for all phosphopeptides identified under these conditions). The phosphorylated residues correspond to serines S281, S284, S343, S345, S346, S349, S358, S359, S427, S431, and S520 in the Mig1 reference sequence (encoded by C5_02940C_A). Due to recently discovered allelic differences between the two *MIG1* alleles of strain SC5314 that are not included in the *Candida* Genome Database (13), the phosphorylated serines correspond to S285, S288, S347, S349, S350, S353, S362, S363, S431, S435, and S524 in the Mig1 protein encoded by allele 2. For convenience, we refer to the positions in the Mig1 reference sequence throughout this study.

**TABLE 1:** Phosphopeptides identified in Mig1

Peptide sequence^*a*^	Modification	Phosphosites in Mig1	PSMs
SSSSTNLAGLQR	1×Phospho [S3(100)]	S358	652
KSRPNSPSQTPIHLSSSR	2×Phospho [S2(100); S6(100)]	S427, S431	509
SESSTSLYSDGNK	1×Phospho [S9(100)]	S284	357
SRPNSPSQTPIHLSSSR	1×Phospho [S5(100)]	S431	357
LFNASSSSLSSLSGK	2×Phospho [S5(100); S8(100)]	S343, S346	234
LFNASSSSLSSLSGK	1×Phospho [S8(100)]	S346	199
SRPNSPSQTPIHLSSSR	2×Phospho [S1(100); S5(100)]	S427, S431	129
SSSSTNLAGLQR	2×Phospho [S3(100); S4(100)]	S358, S359	16
SVLSFTSLVDYPDPK	1×Phospho [S4(100)]	S520	12
LFNASSSSLSSLSGK	3×Phospho [S7(100); S8(100); S11(100)]	S345, S346, S349	9
SESSTSLYSDGNK^*b*^	1×Phospho [S6(99.5)]	S281	9

^*a*^ Phosphorylated serine residues are underlined.

^*b*^ Peptide detected in a preliminary phosphoproteomics study.

### Repressor activity of mutated Mig1 proteins

To investigate the importance of the phosphorylated serine residues for Mig1 function, we generated a set of mutated *MIG1* alleles in which one to three serine codons were changed to alanine codons, which would prevent phosphorylation of the encoded proteins at these sites (see Materials and Methods). For detection of the various Mig1 proteins, a 3× HA tag was fused to their C-terminus.

**Fig 1:**
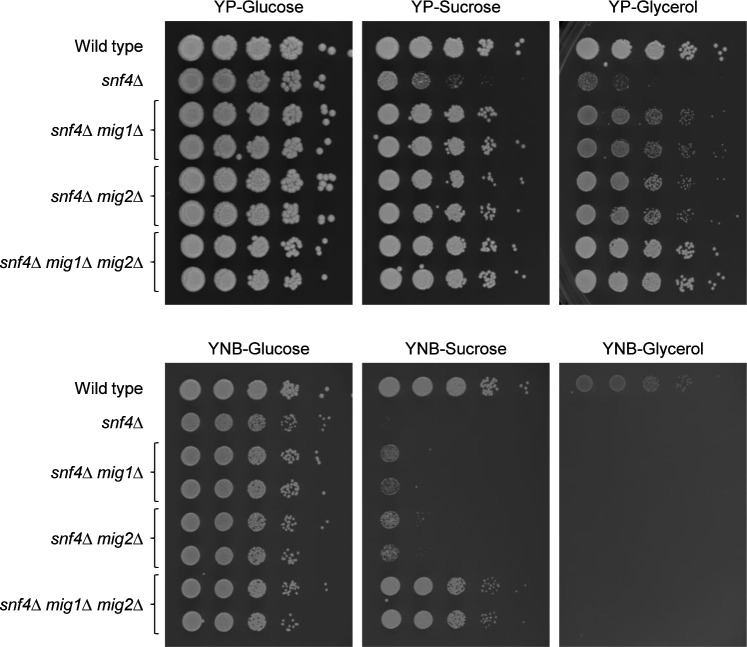
Growth of *snf4*Δ single mutants, *snf4*Δ *mig1*Δ and *snf4*Δ *mig2*Δ double mutants, and *snf4*Δ *mig1*Δ *mig2*Δ triple mutants on different carbon sources. Serial dilutions of strains with the indicated genotypes were spotted on YP or YNB agar plates with 2% glucose, sucrose, or glycerol and incubated for 48 h at 30°C. Results for both independently generated series of double and triple mutants are shown.

We first identified conditions in which the functionality of Mig1 could be tested in a simple phenotypic assay. As reported previously (13), *C. albicans snf4*Δ mutants have a severe growth defect on carbon sources other than glucose, but their growth is strongly improved when both *MIG1* and *MIG2* are additionally deleted. We therefore compared the growth of *snf4*Δ mutants lacking both Mig1 and Mig2 with that of *snf4*Δ mutants lacking only one of the two repressors. As can be seen in Fig. 1, on yeast extract-peptone (YP)-based media containing sucrose or glycerol as the main carbon source, deletion of either *MIG1* or *MIG2* alone restored growth at least partially, while on yeast nitrogen base (YNB)-glycerol, on which also the wild type grew poorly, the growth defect of the *snf4*Δ mutants was not overcome even in the absence of both repressors. In contrast, on YNB-sucrose plates only the triple mutants could grow relatively well. These results suggested that reintroduction of a single functional *MIG1* copy into *snf4*∆ *mig1*∆ *mig2*∆ triple mutants might be sufficient to efficiently suppress growth on YNB-sucrose.

**Fig 2:**
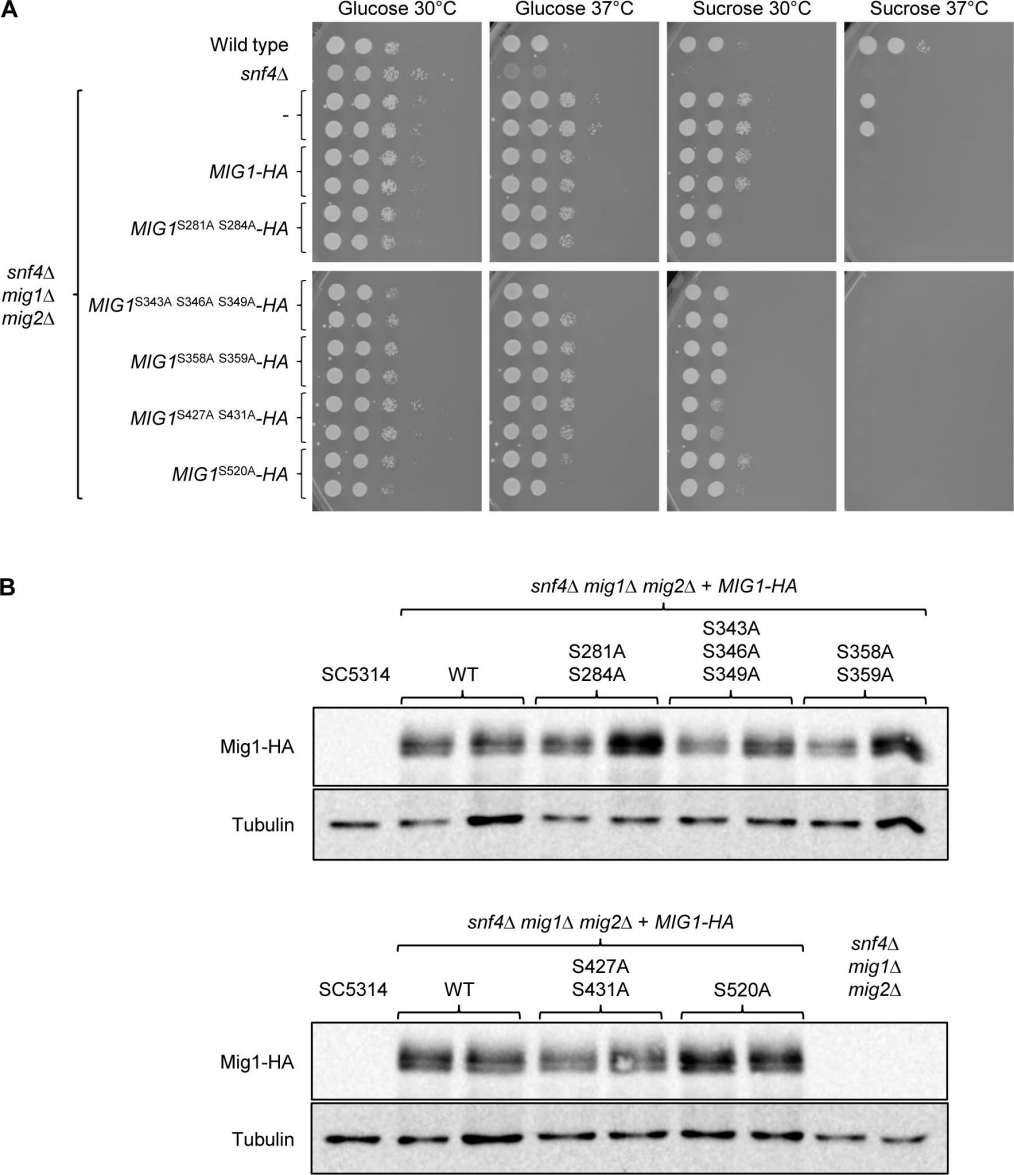
Functionality of mutated Mig1 proteins. (**A**) Serial dilutions of strains with the indicated genotypes were spotted on YNB agar plates with 2% glucose or sucrose and incubated for 48 h at 30°C or 37°C. Strains in the top and bottom panels were grown on the same plate and the photographs arranged accordingly for clarity of presentation. (**B**) Detection of HA-tagged wild-type and mutated Mig1 proteins on western blots with an anti-HA antibody. Cells were grown to log phase in YPD at 30°C. The untagged wild-type strain SC5314 and the parental *snf4*Δ *mig1*Δ *mig2*Δ mutants were used as negative controls. An anti-tubulin antibody was used to control for equal loading. Results for both independently generated series of mutants are shown in (**A**) and (**B**).

**Fig 3:**
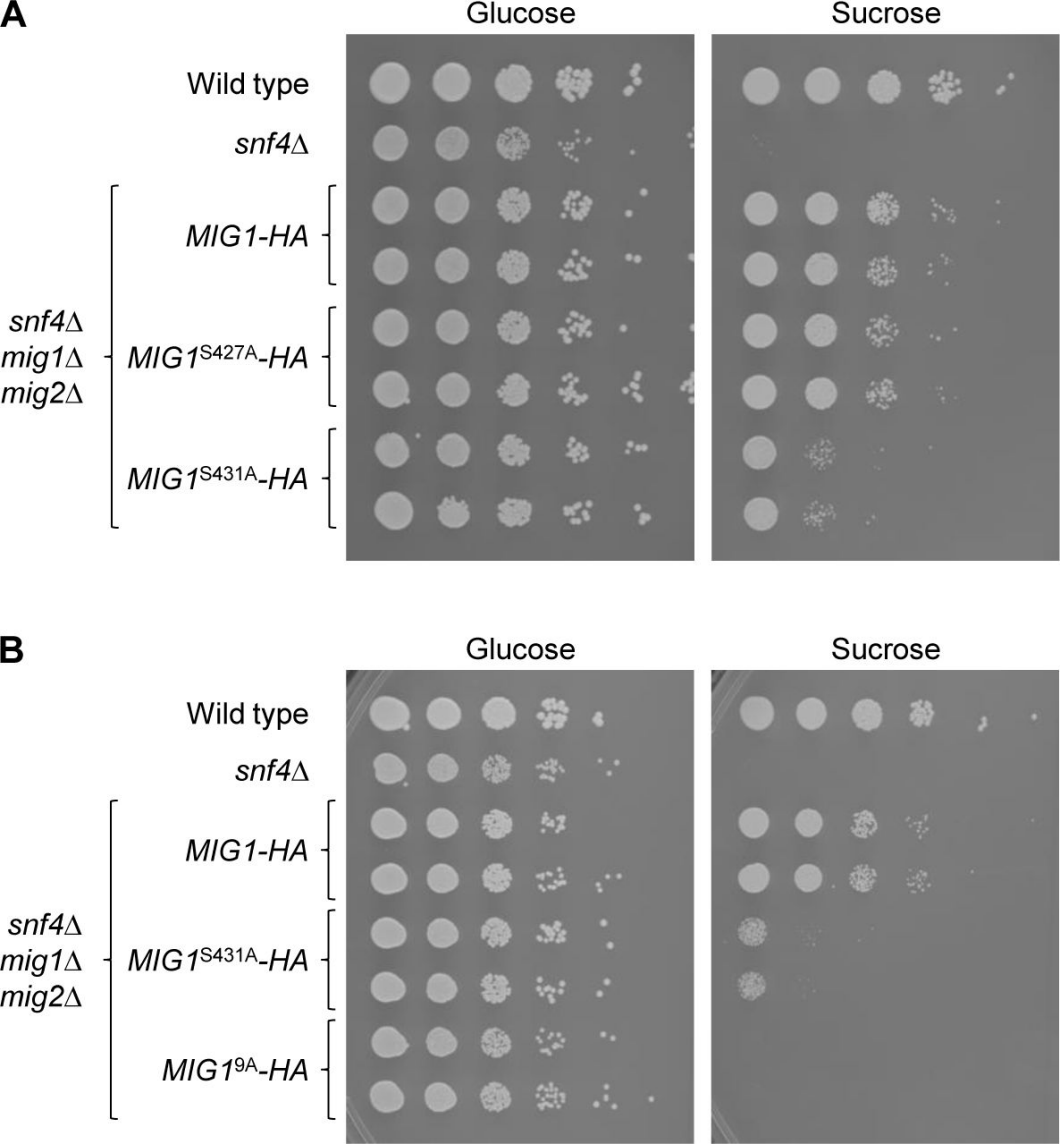
Effect of the S427A, S431A, and 9A mutations on the repressor activity of Mig1. Serial dilutions of strains with the indicated genotypes were spotted on YNB agar plates with 2% glucose or sucrose and incubated for 48 h at 30°C. Results for both independently generated series of mutants are shown.

The HA-tagged wild-type and mutated *MIG1* alleles were integrated at the original locus of two independently generated *snf4*∆ *mig1*∆ *mig2*∆ triple mutants, and growth of the strains was assessed at 30°C and also at 37°C, since *snf4*Δ mutants have a more severe growth defect at the higher temperature even when glucose is the carbon source (13). The absence of both Mig1 and Mig2 restored growth of *snf4*∆ mutants on sucrose at 30°C, but only partially at 37°C (Fig. 2A). Reintroduction of an HA-tagged wild-type *MIG1* copy suppressed growth on sucrose at 37°C, but not at 30°C. All five mutated versions of Mig1 were functional repressors, since they, too, suppressed growth of the triple mutants on sucrose at 37°C. Intriguingly, except for Mig1 with the S520A substitution, the mutated Mig1 proteins also partially suppressed growth on sucrose at 30°C, indicating that they were stronger repressors than wild-type Mig1. Western blotting confirmed that wild-type and mutated proteins were produced in similar amounts (Fig. 2B). The strongest growth suppression was exerted by Mig1 with the S427A and S431A substitutions. To reveal if one or both of these mutations contributed to the effect, we generated *MIG1* alleles with only one of the two codon exchanges. A comparison of the growth of strains containing these *MIG1* versions showed that it was the S431A mutation that turned Mig1 into a stronger growth suppressor (Fig. 3A). In addition, we combined the mutations of the *MIG1*^S281A S284A^, *MIG1*^S343A S346A S349A^, *MIG1*^S358A S359A^, and *MIG1*^S427A S431A^ alleles in a single *MIG1*^9A^ allele. As can be seen in Fig. 3B, Mig1^9A^ was a stronger repressor than Mig1^S431A^, since it completely suppressed growth on sucrose.

### Carbon source regulation of wild-type and mutated Mig1 protein levels

**Fig 4:**
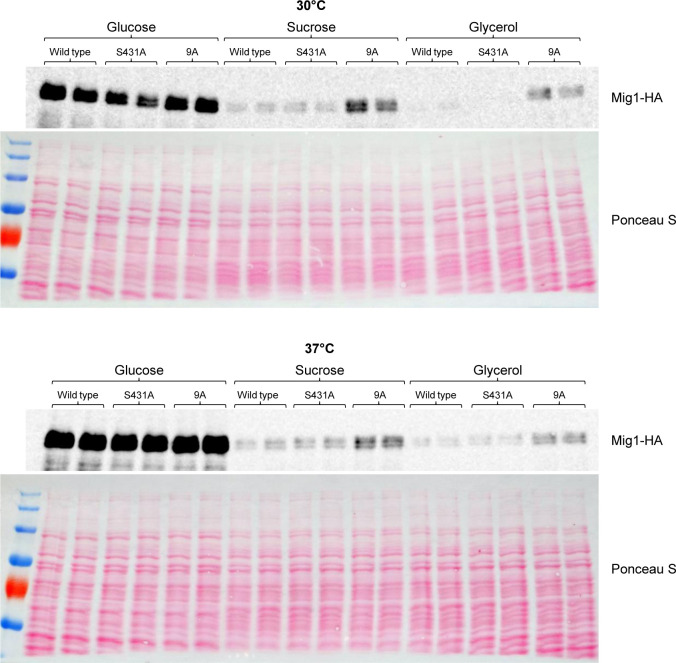
Carbon source regulation of Mig1 protein levels. YPD overnight cultures of *snf4*Δ *mig1*Δ *mig2*Δ mutants containing the indicated HA-tagged *MIG1* alleles were diluted in YNB medium with 2% glucose, sucrose, or glycerol and grown for 3 h at 30°C or 37°C. Mig1 proteins in cell extracts were detected by western blotting with an anti-HA antibody. Ponceau S staining of the blots served as loading control. Results for both independently generated series of mutants are shown.

As explained in the introduction, protein depletion seems to be a mechanism of Mig1 inactivation after shifting the cells from glucose to alternative carbon sources (11, 14). We therefore compared the levels of wild-type Mig1, Mig1^S431A^, and Mig1^9A^ in cells grown on different carbon sources. Interestingly, the levels of all three Mig1 variants were strongly reduced in cells grown on sucrose or glycerol as compared with those in cells grown on glucose, both at 30°C and at 37°C (Fig. 4). In a previous study, we found that Mig1 levels were higher in *snf4*Δ mutants than in the wild type during growth on alternative carbon sources, suggesting that a failure to downregulate Mig1 levels explains the inability of the mutants to grow under these conditions (14). The results shown in Fig. 4 demonstrate that even cells lacking a functional SNF1 complex are still able to strongly reduce Mig1 levels. While the levels of Mig1^9A^ were higher than those of wild-type Mig1 during growth on sucrose or glycerol, there were no observable differences in the levels of wild-type Mig1 and Mig1^S431A^ (Fig. 4), although the latter was a more efficient growth suppressor (see Fig. 3). This finding argues that the mutated Mig1 proteins are stronger repressors than wild-type Mig1.

### Untagged Mig1 proteins exhibit increased repressor activity

**Fig 5:**
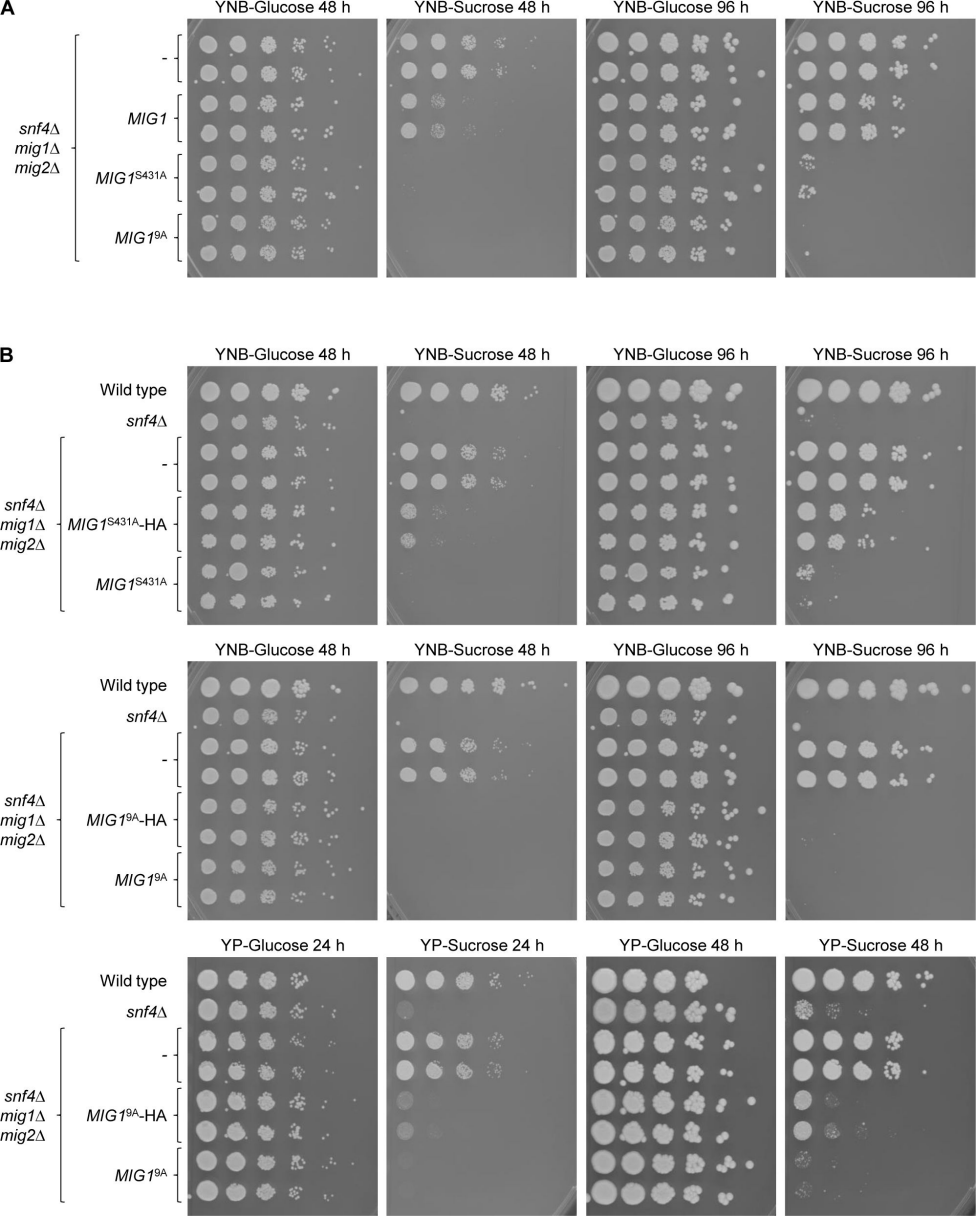
The 3×HA tag has a negative effect on Mig1 activity. Serial dilutions of strains with the indicated genotypes were spotted on YNB or YP agar plates with 2% glucose or sucrose and incubated for the indicated times at 30°C. Results for both independently generated series of mutants are shown. (**A**) Comparison of the effects of wild-type *MIG1*, *MIG1*^S431A^, and *MIG1*^9A^ alleles without the 3×HA tag. (**B**) Direct comparison of 3×HA-tagged and untagged versions of *MIG1*^S431A^ and *MIG1*^9A^.

To exclude the possibility that the mutations only affected the C-terminally 3×HA-tagged Mig1, we reintroduced untagged wild-type *MIG1*, *MIG1*^S431A^, and *MIG1*^9A^ alleles into the *snf4*∆ *mig1*∆ *mig2*∆ triple mutants and compared growth of the resulting strains. Interestingly, a single copy of wild-type *MIG1* suppressed growth of the triple mutants on sucrose also at 30°C, demonstrating that the 3×HA tagging negatively affected the ability of Mig1 to act as a repressor (Fig. 5A, compare with Fig. 2A), which could be caused by impairing protein function but also reducing mRNA stability or translational efficiency. Nevertheless, the S431A mutation also increased the repressor activity of untagged Mig1, and Mig1^9A^ suppressed growth on sucrose even more, although the difference to Mig1^S431A^ became apparent only after prolonged incubation when residual growth of cells containing Mig1^S431A^, but not of cells with Mig1^9A^, could be observed (Fig. 5A, right panel). The 3×HA tag not only affected wild-type Mig1 but also reduced the repressor activity of Mig1 with the S431A and 9A mutations, as can be seen in the direct comparison of 3×HA-tagged and untagged Mig1^S431A^ and Mig1^9A^ (Fig. 5B). In the latter case, a difference could only be detected on YP-sucrose plates (Fig. 5B, bottom panels) on which the strains grow better, because both tagged and untagged Mig1^9A^ completely suppressed growth of the triple mutants on YNB-sucrose (Fig. 5B, middle panels).

### A functional SNF1 complex can still inhibit the hyperactive Mig1^9A^

**Fig 6:**
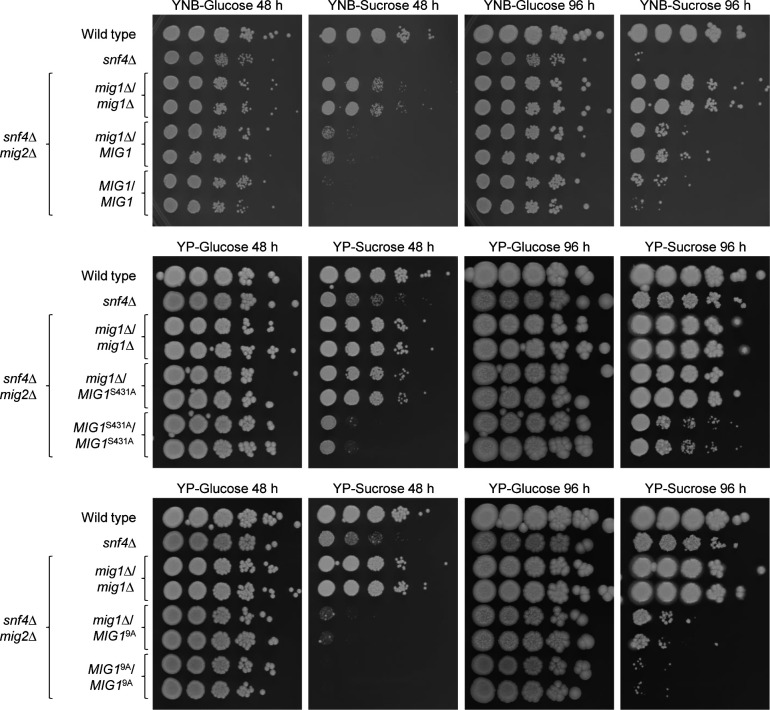
*MIG1* homozygosity increases repressor activity. Serial dilutions of strains with the indicated genotypes were spotted on YNB or YP agar plates with 2% glucose or sucrose and incubated for the indicated times at 30°C. Results for both independently generated series of mutants are shown.

So far, the activities of wild-type and mutated Mig1 proteins were compared in the absence of a functional SNF1 complex (in an *snf4*Δ background). We wondered if Mig1^S431A^ and Mig1^9A^ could suppress growth even in the presence of SNF1, i.e., if SNF1 would fail to adequately inhibit the mutated repressors. We first tested if reintroduction of two instead of only one copy of wild-type and mutated *MIG1* alleles into the *snf4*∆ *mig1*∆ *mig2*∆ triple mutants further increased their ability to inhibit growth on sucrose. As expected, this was the case for all three *MIG1* variants. For wild-type *MIG1*, this was clearly observable on YNB-sucrose plates (Fig. 6, top panels). For *MIG1*^S431A^ and *MIG1*^9A^, where a single copy was sufficient to (almost) completely suppress growth on YNB-sucrose (see Fig. 5A), the dosage effect became evident on YP-sucrose plates (Fig. 6, middle and bottom panels). We then introduced wild-type *MIG1*, *MIG1*^S431A^, and *MIG1*^9A^ alleles into the original *MIG1* locus on both homologous chromosomes of *mig1*∆ *mig2*∆ mutants and compared the growth phenotypes of the resulting strains. In addition, one copy of the 3×HA-tagged versions of the different *MIG1* alleles was introduced into the same mutants to compare protein levels.

**Fig 7:**
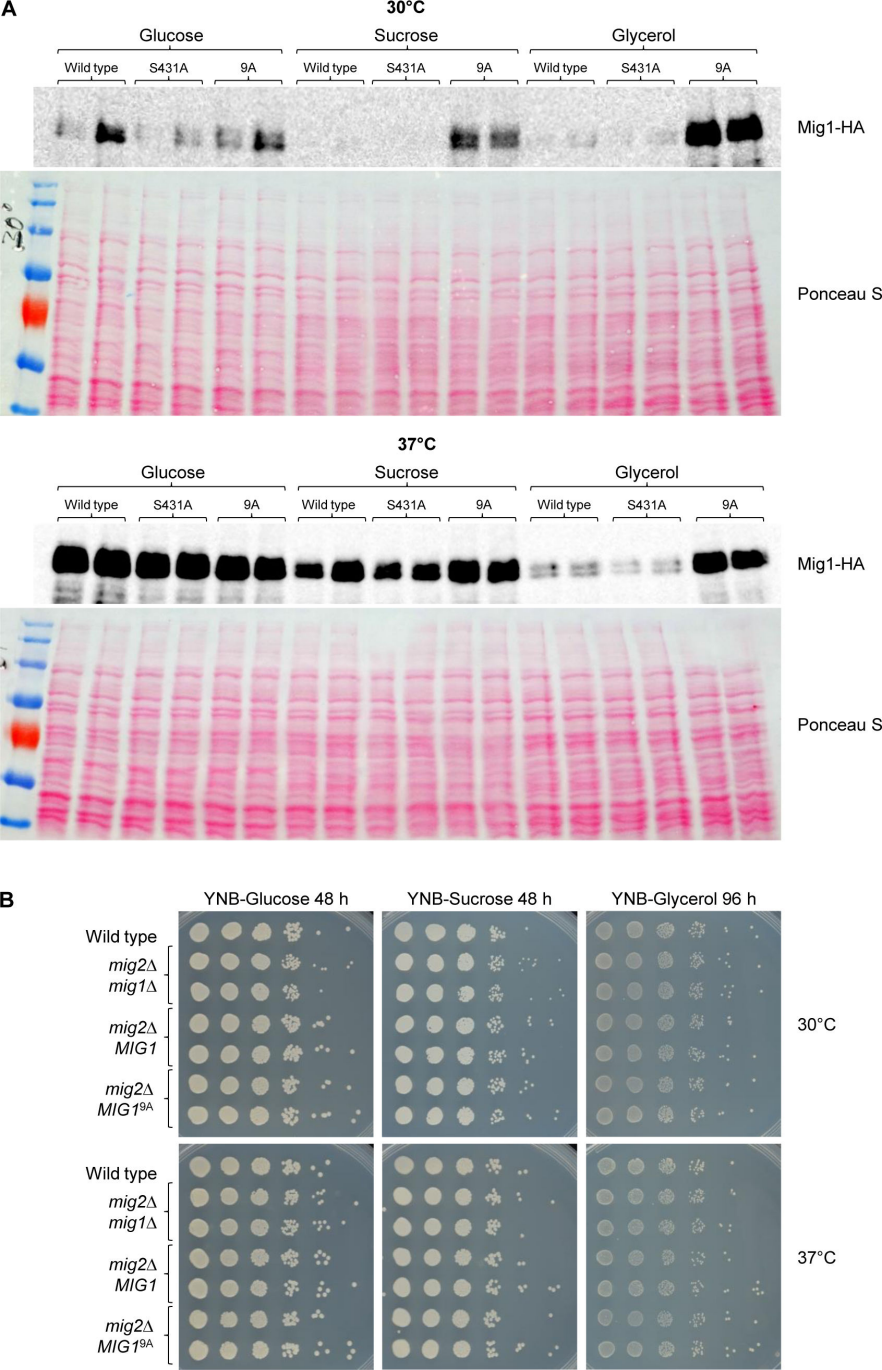
A functional SNF1 complex can inhibit the mutated Mig1 proteins. (**A**) YPD overnight cultures of *mig1*Δ *mig2*Δ mutants containing the indicated HA-tagged *MIG1* alleles were diluted in YNB medium with 2% glucose, sucrose, or glycerol and grown for 3 h at 30°C or 37°C. Mig1 proteins in cell extracts were detected by western blotting with an anti-HA antibody. Ponceau S staining of the blots served as loading control. (**B**) Serial dilutions of the wild-type strain SC5314, *mig1*Δ *mig2*Δ mutants, and derivatives containing untagged wild-type *MIG1* or *MIG1*^9A^ alleles on both chromosomes were spotted on YNB agar plates with 2% glucose, sucrose, or glycerol and grown for 48 or 96 h at 30°C or 37°C. Results for both independently generated series of mutants are shown in (**A**) and (**B**).

Figure 7A shows the levels of the Mig1 proteins in cells grown on glucose, sucrose, or glycerol as carbon source. For unknown reasons, the levels of wild-type Mig1 differed in two independent transformants under some conditions. Irrespective of this, the results show that the levels of Mig1^S431A^ were similar to those of wild-type Mig1 under all conditions, whereas the levels of Mig1^9A^ were strongly increased compared with those of wild-type Mig1 during growth on sucrose at 30°C and when glycerol was the carbon source. We therefore compared the growth of *mig2*Δ strains lacking *MIG1* or containing two copies of wild-type *MIG1* or *MIG1*^9A^. Figure 7B shows that despite its increased levels, Mig1^9A^ was unable to suppress growth in media with sucrose or glycerol as the sole carbon source, i.e., even this hyperactive repressor could efficiently be inhibited in the presence of a functional SNF1 complex.

## DISCUSSION

The well-established functions of the protein kinase SNF1 and its downstream target Mig1 in regulating the utilization of alternative carbon sources are conserved in *C. albicans*, but the mechanism of how Mig1 activity is controlled in this species has remained elusive so far (11, 13, 14). Here, by harnessing data from a phosphoproteomics analysis of the wild-type strain SC5314 and mutant derivatives, we have identified several phosphorylation sites in Mig1. Previous work has shown that Mig1 is phosphorylated also during growth in glucose-rich medium, although Mig1 is expected to repress expression of its target genes and therefore not be inhibited under these conditions (14). The results of our present study demonstrate that at least four of the identified phosphorylation sites (and probably more, since we did not mutate all phosphorylated serines individually and in all possible combinations) contribute to the inhibition of Mig1 during growth on sucrose as an alternative carbon source (see Fig. 2A). While we cannot exclude the possibility that the change of a polar amino acid (serine) to a nonpolar amino acid (alanine) turned Mig1 into a stronger repressor, the fact that the serines were found to be phosphorylated in the phosphoproteomics analysis argues that Mig1 activity is reduced by phosphorylation at these sites. Therefore, some negative regulation of Mig1 seems to occur even in YPD medium to allow a simultaneous utilization of carbon sources other than glucose. In line with this, the expression of many genes with metabolic functions was decreased under these conditions in mutants lacking the SNF1-activating kinase Sak1, presumably due to unrestricted Mig1 (and Mig2) activity (3).

Previous work has shown that Mig1 levels are decreased when *C. albicans* grows on alternative carbon sources compared with growth on glucose, indicating that protein depletion is a mechanism to inactivate the repressor (11, 14). Our results suggest that Mig1 phosphorylation reduces its repressor activity also independently of protein levels. The Mig1^S431A^ variant, which lacks one of the identified phosphorylation sites, was present in comparable amounts to those of wild-type Mig1 and similarly downregulated during growth on sucrose or glycerol (Fig. 4) but nevertheless suppressed growth on sucrose more efficiently than wild-type Mig1 in the absence of a functional SNF1 complex (Fig. 3A and 5A). Furthermore, the levels of the Mig1^9A^ variant, which lacks nine phosphorylation sites and could strongly suppress growth on sucrose in the absence of SNF1 (Fig. 5), were strongly increased compared with those of wild-type Mig1 even in cells with a functional SNF1 complex, especially during growth on glycerol (Fig. 7A). In spite of this, Mig1^9A^ was unable to suppress growth in these cells (Fig. 7B), indicating that SNF1 could still inactivate Mig1 even when its levels were not reduced and even when nine phosphorylation sites were missing. In fact, we did not observe differences in the migration patterns of wild-type Mig1 and any of the variants lacking specific phosphorylation sites, including Mig1^9A^, in the western blots. Previous work has shown that the Mig1 forms with reduced mobility disappeared when the samples were treated with phosphatase (14). This argues that Mig1 contains additional phosphorylation sites that were not detected in our phosphoproteome analysis and some of these are critical to inactivate the repressor by SNF1-dependent phosphorylation. Of note, Mig1^9A^ forms with reduced mobility were also observed in *snf4*Δ mutants (Fig. 4), pointing to the involvement of additional kinase(s) in the regulation of Mig1, as suggested previously (14). These kinases may reduce Mig1 activity to some extent independently of SNF1, as suggested by the observation that loss of several of the identified phosphorylation sites increased Mig1 repressor activity in the absence of SNF1 (Fig. 2A, 3 and 5A). Screening our protein kinase deletion mutant library (16) did not discover mutants that had a growth defect on sucrose but grew normally on glucose, presumably because of the presence of SNF1 in these mutants. Therefore, which protein kinases directly phosphorylate Mig1 and how SNF1 regulates their activity remain to be established.

## MATERIALS AND METHODS

### Strains and growth conditions

The *C. albicans* strains used in this study are listed in Table S2. All strains were stored as frozen stocks with 17.2% glycerol at −80°C and subcultured on YPD agar plates (10 g yeast extract, 20 g peptone, 20 g glucose, and 15 g agar per liter) at 30°C. Strains were routinely grown in YPD liquid medium at 30°C in a shaking incubator. For the selection of transformants, 200 µg/mL nourseothricin (Werner Bioagents) was added to YPD agar plates. To obtain nourseothricin-sensitive derivatives in which the *SAT1* flipper cassette was excised by FLP-mediated recombination, transformants were grown overnight in YCB-BSA-YE medium (23.4 g yeast carbon base, 4 g bovine serum albumin, and 2 g yeast extract per liter, pH 4.0) without selective pressure to induce the *SAP2* promoter controlling *caFLP* expression. Appropriate dilutions were plated on YPD agar plates and grown for 2 days at 30°C. Individual colonies were picked and streaked on YPD plates as well as on YPD plates with 100 µg/mL nourseothricin to confirm sensitivity.

### Plasmid constructions

To generate 3×HA-tagged *MIG1* alleles, the *MIG1* coding region and upstream sequences (allele 2) were amplified from genomic DNA of strain SC5314 with primers MIG1.01 and MIG1.05; the latter primer introduces a KasI site, encoding a Gly-Ala linker, instead of the *MIG1* stop codon (all primers used in this study are listed in Table S3). A fragment from plasmid pMIG1H1 (14), encoding three copies of the HA epitope followed by a stop codon and the *ACT1* transcription termination sequence, was amplified with primers HAT7 and ACT19. The two PCR products were digested with SacI/KasI and KasI/SacII, respectively, and ligated together in the SacI/SacII-digested pMIG1H1 to obtain pMIG1H3. The 3×HA-tagged *MIG1*^S281A S284A^ allele was generated by a fusion PCR with the primer pairs MIG1.01/MIG1H4R and MIG1H4F/MIG1.05 using pMIG1H3 as template; the overlapping primers MIG1H4R and MIG1H4F change the codons for Ser281 and Ser284 into alanine codons. The PCR product was digested with SacI/KasI and substituted for the wild-type *MIG1* sequence in pMIG1H3 to obtain pMIG1H4. The 3×HA-tagged *MIG1*^S343A S346A S349A^, *MIG1*^S358A S359A^, *MIG1*^S427A S431A^, *MIG1*^S427A^, *MIG1*^S431A^, and *MIG1*^S520A^ alleles were obtained in an analogous fashion using the mutagenic primer pairs MIG1H5R/MIG1H5F, MIG1H6R/MIG1H6F, MIG1H7R/MIG1H7F, MIG1H7aR/MIG1H7aF, MIG1H7bR/MIG1H7bF, and MIG1H8R/MIG1H8F to generate plasmids pMIG1H5, pMIG1H6, pMIG1H7, pMIG1H7a, pMIG1H7b, and pMIG1H8, respectively. The S281A to S431A mutations were also combined in sequential fusion PCRs with appropriate primer pairs to obtain the *MIG1*^9A^ allele in the final plasmid pMIG1H11A. An untagged wild-type copy of *MIG1* allele 2 including 0.5 kb of upstream and downstream sequences was amplified with the primers MIG1.01 and MIG1.06. The PCR product was digested with SacI/SacII and substituted for the HA-tagged *MIG1* in pMIG1H3 to obtain pMIG1K1. The S431A mutation was introduced by a fusion PCR with the primer pairs MIG1.01/MIG1H7bR and MIG1H7bF/MIG1.06 and substituting the resulting *MIG1*^S431A^ allele for the wild-type allele in pMIG1K1, yielding pMIG1K2. To obtain an untagged version of the *MIG1*^9A^ allele, pMIG1H11 and pMIG1K1 served as templates for PCRs with the primer pairs MIG1.05/MIG1H7R and MIG1H7F/MIG1.06, respectively, followed by a fusion PCR with primers MIG1.01 and MIG1.06 and substituting the SacI/SacII-digested PCR product for the wild-type allele in pMIG1K1, yielding pMIG1K3.

### Strain constructions

*C. albicans* strains were transformed by electroporation (17) with the gel-purified inserts from the plasmids described above to express the various *MIG1* alleles in *snf4*∆ *mig1*∆ *mig2*∆ triple mutants (13) and in *mig1*∆ *mig2*∆ double mutants. The *mig1*∆ *mig2*∆ double mutants were generated by sequentially deleting the *MIG1* and *MIG2* alleles in the wild-type strain SC5314 with the inserts from plasmids pMIG1M1 and pMIG2M1 (13). To compare the effect of *MIG2* deletion with the effects of deletion of *MIG1* or both *MIG1* and *MIG2* in an *snf4*Δ background, the *MIG2* alleles were also sequentially deleted in *snf4*Δ mutants. The correct genomic integration of all constructs and subsequent excision of the *SAT1* flipper cassette were confirmed by southern hybridization using the flanking sequences as probes. The other strains used in this study have been described previously (see Table S2).

### Isolation of genomic DNA and southern hybridization

Genomic DNA from *C. albicans* strains was isolated as described previously (18). The DNA was digested with appropriate restriction enzymes, separated on a 1% agarose gel, transferred by vacuum blotting onto a nylon membrane, and fixed by UV crosslinking. Southern hybridization with enhanced chemiluminescence-labeled probes was performed with the Amersham ECL Direct Nucleic Acid Labelling and Detection System (Cytiva) according to the instructions of the manufacturer.

### Growth assays

To compare growth of the *C. albicans* strains on different carbon sources, YPD overnight cultures were adjusted to an optical density at 600 nm (OD_600_) of 2.0 in water, serially 10-fold diluted, and spotted on YP (1% yeast extract, 2% peptone, and 1.5% agar) or YNB (0.67% yeast nitrogen base with ammonium sulfate, 2% agar) plates containing 2% glucose, sucrose, or glycerol as carbon source. Plates were incubated for 1–4 days at 30°C or 37°C.

### Western blotting

YPD overnight cultures of the strains were diluted to an OD_600_ of 0.6 in YNB medium containing 2% glucose, sucrose, or glycerol and grown for 3 h at 30°C or 37°C. Cells were collected by centrifugation, washed with ice-cold water, and, depending on pellet volume, resuspended in 100 to 300 µL breaking buffer (50 mM Tris-HCl, pH 8, 250 mM NaCl, 5 mM EDTA, 0.1% [vol/vol] Triton X-100, cOmplete EDTA-free Protease Inhibitor Cocktail and PhosStop Phosphatase Inhibitor Cocktail [Roche]). An equal volume of 0.5 mm acid-washed glass beads was added to each tube. Cells were mechanically disrupted on a FastPrep-24 cell-homogenizer (MP Biomedicals) with three 40-s pulses, with 5 min on ice between each pulse. Cell lysates were centrifuged at 21,000 × *g* for 15 min at 4°C, the supernatant was collected, and the protein concentration was quantified using the Bradford protein assay. Equal amounts of protein of each sample were mixed with one volume of 2× Laemmli buffer, heated for 5 min at 95°C, and separated on an SDS-8% polyacrylamide gel. Separated proteins were transferred onto a nitrocellulose membrane with a semi-dry blotter (Owl). To detect HA-tagged Mig1 proteins, membranes were blocked with 5% milk in Tris-buffered saline with Tween 20 (TBST) and incubated overnight with rat monoclonal anti-HA-peroxidase antibody, clone 3F10 (Roche). For the detection of tubulin, membranes were blocked with 5% milk in TBST and incubated overnight at 4°C with rat anti-tubulin alpha antibody MCA 78G (Bio-Rad), washed with TBST, and then incubated with rabbit anti-rat HRP-conjugated antibody STAR21B (Bio-Rad). To reprobe the immunoblots, membranes were incubated in stripping buffer (0.2 M glycine, 0.1% SDS, 1% Tween 20, pH 2.2) and washed in phosphate-buffered saline (PBS) and TBST before blocking with 5% milk. Signals were generated with the ECL chemiluminescence detection system (Cytiva) and captured with the ImageQuant LAS 4000 imaging system (Cytiva).

### Phosphoproteome analysis

Cells were harvested by centrifugation and mechanically disrupted with a mortar and pestle in the presence of liquid nitrogen. Cell debris was homogenized in lysis buffer (100  mM TEAB, 150  mM NaCl, 1% SDS, cOmplete Ultra EDTA-free and PhosStop [both Roche Diagnostics GmbH]). Samples were further incubated with 250 units benzonase (Merck Millipore, Billerica, MA, USA) for 30 min in a water bath sonicator at 37°C. Proteins were separated from unsolubilized debris by centrifugation (15 min, 18,000 × *g*). Each 1.5 mg of total protein per sample was diluted with 100 mM TEAB to gain a final volume of 1.5 mL. Subsequently, cysteine thiols were reduced and carbamidomethylated in one step for 30 min at 70°C by the addition of 30 µL of 500 mM TCEP [tris(2-carboxyethyl)phosphine] and 30 µL of 625 mM 2-chloroacetamide (CAA). The samples were further cleaned up by 20% trichloroacetic acid (20 min, 20,000 × *g*, 4°C) and washed with 90% acetone (20 min, 20,000 × *g*, 4°C). Protein precipitates were resolubilized in 5% trifluoroethanol of aqueous 100 mM TEAB and digested overnight (18 h) with a Trypsin + LysC mixture (Promega) at a protein to protease ratio of 25:1. Each sample was divided in 3 × 0.5 mg used for the phosphopeptide enrichment and 150 µg initial protein used for the reference proteome analysis. Samples were evaporated in a vacuum concentrator (Eppendorf). The reference proteome sample was resolubilized in 30 µL of 0.05% trifluoroacetic acid (TFA) in H_2_O/acetonitrile (ACN) 98/2 (vol/vol) filtered through 10-kDa MWCO PES membrane spin filters (VWR). The filtrate was transferred to HPLC vials and injected into the liquid chromatography tandem mass spectrometry (LC-MS/MS) instrument.

Phosphopeptides were enriched by using TiO_2_ + ZrO_2_ TopTips (Glygen Corp.). TopTips were loaded with 0.5 mg protein isolate using three TopTips per biological replicate after equilibration with 200 µL Load and Wash Solution 1(LWS1; 1% TFA, 20% lactic acid, 25% ACN, and 54% H_2_O). TopTips were centrifuged at 200 × *g* for 5 min at room temperature. After washing with 200 µL LWS1, the TiO_2_/ZrO_2_ resin was washed with 25% ACN, and subsequently, the phosphopeptides were eluted with 200 µL NH_3_ · H_2_O (NH_4_OH), pH 12. The alkaline solution was immediately evaporated using a vacuum concentrator (Eppendorf). The phosphoproteome samples were resolubilized in 50 µL of 0.05% TFA in H_2_O/ACN 98/2 (vol/vol) filtered through 10-kDa MWCO PES membrane spin filters (VWR). The filtrate was also transferred to HPLC vials and injected into the LC-MS/MS instrument.

Each sample was measured in duplicate (two analytical replicates of three biological replicates of a reference proteome fraction and a phosphoproteome fraction). LC-MS/MS analysis was performed on an Ultimate 3000 nano RSLC system connected to a QExactive HF mass spectrometer (both Thermo Fisher Scientific, Waltham, MA, USA). Peptide trapping for 5 min on an Acclaim Pep Map 100 column (2 cm × 75 µm, 3 µm) at 5 µL/min was followed by separation on an analytical Acclaim Pep Map RSLC nano column (50 cm × 75 µm, 2 µm). Mobile phase gradient elution of eluent A [0.1% (vol/vol) formic acid in water] mixed with eluent B (0.1% [vol/vol] formic acid in 90/10 acetonitrile/water) was performed using the following gradient: 0–5 min at 4% B, 30 min at 7% B, 60 min at 10% B, 100 min at 15% B, 140 min at 25% B, 180 min at 45% B, 200 min at 65% B, 210–215 min at 96% B, and 215.1–240 min at 4% B. Positively charged ions were generated at a spray voltage of 2.2 kV using a stainless steel emitter attached to the Nanospray Flex Ion Source (Thermo Fisher Scientific). The quadrupole/orbitrap instrument was operated in Full MS/data-dependent MS2 Top15 mode. Precursor ions were monitored at m/z 300–1,500 at a resolution of 120,000 FWHM (full width at half maximum) using a maximum injection time (ITmax) of 120 ms and an AGC (automatic gain control) target of 3 × 10^6^. Precursor ions with a charge state of *z* = 2–5 were filtered at an isolation width of *m/z* 1.6 amu for further HCD fragmentation at 27% normalized collision energy. MS2 ions were scanned at 15,000 FWHM (ITmax = 100 ms, AGC = 2 × 10^5^) using a fixed first mass of *m/z* 120 amu. Dynamic exclusion of precursor ions was set to 30 s, and the minimum AGC target for precursor ions selected for HCD fragmentation was set to 1 × 10^3^. The LC-MS/MS instrument was controlled by Chromeleon 7.2, QExactive HF Tune 2.8 and Xcalibur 4.0 software.

Tandem mass spectra were searched against the CGD database available at http://www.candidagenome.org/download/sequence/C_albicans_SC5314/Assembly22/current/C_albicans_SC5314_A22_current_orf_trans_all.fasta.gz (22 April 2021) using Proteome Discoverer (PD) 2.4 (Thermo) and the algorithms of Mascot v2.4.1 (Matrix Science, UK), Sequest HT, MS Amanda 2.0, and MS Fragger 2.4. Two missed cleavages were allowed for the tryptic digestion. The precursor mass tolerance was set to 10 ppm, and the fragment mass tolerance was set to 0.02 Da. Modifications were defined as dynamic Met oxidation; phosphorylation of Ser, Thr, and Tyr; protein N-term acetylation with and without Met-loss; and static Cys carbamidomethylation. A strict false discovery rate (FDR) < 1% (peptide and protein levels) and an ion score (Mascot) > 30 or an Xcorr score > 4 (Sequest) or an MS Amanda score > 300 or an MS Fragger score > 8 was required for positive protein hits. The Percolator node of PD2.4 and a reverse decoy database were used for *q*-value validation of spectral matches. Only rank 1 proteins and peptides of the top-scored proteins were counted. Label-free protein quantification was based on the Minora algorithm of PD2.4 using the precursor abundance based on intensity and a signal-to-noise ratio > 5. Normalization was performed by using the total peptide amount method. Imputation of missing quan values was applied by using abundance values of 75% of the lowest abundance identified per sample. For the reference proteome analysis used for master protein abundance correction of the phosphoproteome data, phosphopeptides were excluded from quantification. Differential protein and phosphopeptide abundance were defined as a fold change of >2, *P* value/ABS(log4ratio) < 0.05 and at least identified in two of three replicates of the sample group with the highest abundance.

The mass spectrometry proteomics data have been deposited to the ProteomeXchange Consortium via the PRIDE (19) partner repository with the data set identifier PXD045393.

## Data Availability

The mass spectrometry proteomics data have been deposited to the ProteomeXchange Consortium via the PRIDE (19) partner repository with the data set identifier PXD045393.

## SUPPLEMENTAL MATERIAL

Table S1 msphere.00546-23-s0001.xlsx Identified phosphopeptides.

Table S2 msphere.00546-23-s0002.xlsx *C. albicans* strains used in this study.

Table S3 msphere.00546-23-s0003.xlsx Oligonucleotide primers used in this study.

ASM does not own the copyrights to Supplemental Material that may be linked to, or accessed through, an article. The authors have granted ASM a non-exclusive, world-wide license to publish the Supplemental Material files. Please contact the corresponding author directly for reuse.
